# Deep targeted sequencing of 12 breast cancer susceptibility regions in 4611 women across four different ethnicities

**DOI:** 10.1186/s13058-016-0772-7

**Published:** 2016-11-05

**Authors:** Sara Lindström, Akweley Ablorh, Brad Chapman, Alexander Gusev, Gary Chen, Constance Turman, A. Heather Eliassen, Alkes L. Price, Brian E. Henderson, Loic Le Marchand, Oliver Hofmann, Christopher A. Haiman, Peter Kraft

**Affiliations:** 1 Department of Epidemiology, University of Washington, 1959 N.E. Pacific Street, Health Sciences Building, Room F247B, Seattle, WA 98195 USA; 2 Department of Epidemiology, Harvard T.H. Chan School of Public Health, Boston, MA 02115 USA; 3 Department of Biostatistics, Harvard T.H. Chan School of Public Health, Boston, MA 02115 USA; 4 HSPH Bioinformatics Core, Harvard T.H. Chan School of Public Health, Boston, MA 02115 USA; 5 Department of Preventive Medicine, Norris Comprehensive Cancer Center, Keck School of Medicine, University of Southern California, Los Angeles, CA 90033 USA; 6 Channing Division of Network Medicine, Department of Medicine, Brigham and Women’s Hospital and Harvard Medical School, Boston, MA 02115 USA; 7 Cancer Research Center of Hawai’i, University of Hawai’i, Honolulu, HI 96813 USA

**Keywords:** Breast cancer, Fine-mapping, Next-generation sequencing, Multiethnic analysis, GWAS

## Abstract

**Background:**

Although genome-wide association studies (GWASs) have identified thousands of disease susceptibility regions, the underlying causal mechanism in these regions is not fully known. It is likely that the GWAS signal originates from one or many as yet unidentified causal variants.

**Methods:**

Using next-generation sequencing, we characterized 12 breast cancer susceptibility regions identified by GWASs in 2288 breast cancer cases and 2323 controls across four populations of African American, European, Japanese, and Hispanic ancestry.

**Results:**

After genotype calling and quality control, we identified 137,530 single-nucleotide variants (SNVs); of those, 87.2 % had a minor allele frequency (MAF) <0.005. For SNVs with MAF >0.005, we calculated the smallest number of SNVs needed to obtain a posterior probability set (PPS) such that there is 90 % probability that the causal SNV is included. We found that the PPS for two regions, 2q35 and 11q13, contained less than 5 % of the original SNVs, dramatically decreasing the number of potentially causal SNVs. However, we did not find strong evidence supporting a causal role for any individual SNV. In addition, there were no significant gene-based rare SNV associations after correcting for multiple testing.

**Conclusions:**

This study illustrates some of the challenges faced in fine-mapping studies in the post-GWAS era, most importantly the large sample sizes needed to identify rare-variant associations or to distinguish the effects of strongly correlated common SNVs.

**Electronic supplementary material:**

The online version of this article (doi:10.1186/s13058-016-0772-7) contains supplementary material, which is available to authorized users.

## Background

Breast cancer is the most common malignancy among women in the United States, with more than 230,000 new diagnoses expected in 2015 [1]. Breast cancer has a heritable component [2], and researchers in recent genome-wide association studies (GWASs) have identified more than 90 [3–20] genetic regions associated with breast cancer risk. However, the underlying causal mechanism in these regions is not fully known, and it is likely that the index GWAS signal originates from one or many as yet unidentified causal variants. Because GWASs rely on linkage disequilibrium (LD) or correlation between neighboring common genetic variants, they cannot be used to localize causal variants with precision. Instead, one genetic variant is used to tag segments of the genome over which LD is maintained (“LD blocks”), which can contain multiple genes. Localizing causal variation is further complicated by the possibility of multiple causal variants within one tagged segment of the genome.

Moreover, GWAS-identified regions that contain common risk variants may also contain rare variations associated with disease risk. For example, rare susceptibility variants for ulcerative colitis [21] and inflammatory bowel disease [22] have been identified in GWAS regions. In principle, GWAS-identified risk single-nucleotide polymorphisms (SNPs) may be proxies for multiple rare risk alleles (“synthetic association”) [23]. Indeed, dense genotyping of the *HOXB* region revealed a cluster of common, low-penetrance prostate cancer risk alleles that appear to tag the rare, moderate-penetrance coding variant rs138213197 [24]. However, in practice, the LD between common SNPs on GWAS platforms and rare variants is low. This implies that direct measurement of rare variants is needed to identify rare-variant associations at GWAS-identified loci. Fine-mapping of GWAS-identified regions with the aim of identifying and prioritizing causal variants requires not only large sample sizes but also a comprehensive capture of the genetic variation, with the latter often not achieved through standard GWAS arrays. Sequencing is an attractive approach, but until recently it has been prohibitively expensive to do on a large-scale basis. Early fine-mapping studies sequenced a small number of cases and then genotyped detected SNPs in a larger population [22, 25]. Like GWAS, however, this approach will likely miss rare variants because of the low number of subjects initially sequenced.

A limitation in previous breast cancer studies is the lack of well-powered studies across multiple ancestral populations. Although breast cancer GWASs have been conducted in populations of Asian [5, 11, 12, 19, 20], African [26], and Hispanic [27] ancestry, the vast majority of studies have been conducted in European [3, 4, 6–8, 10, 13, 14, 16–18] ancestry populations, and only a few GWASs have been conducted across ethnicities [9, 15]. LD blocks differ by ancestry, which limits the consistency of some GWAS findings across populations, and studies with subjects of a single ethnicity may miss risk alleles that are observed at higher frequencies in other populations [28]. Indeed, multiethnic studies of genetic susceptibility regions discovered in a specific ethnicity often identify different “top” variants across ethnicities [29–33]; therefore, multiethnic studies have been proposed to aid fine-mapping of causal variants [28]. In this study, we attempted to overcome many of the issues related to fine-mapping of GWAS regions by using next-generation sequencing to characterize 12 breast cancer susceptibility regions in a multiethnic sample of 2288 breast cancer cases and 2323 controls.

## Methods

### Study subjects

The Nurses’ Health Study (NHS) was initiated in 1976, when 121,700 U.S. registered nurses aged 30 to 55 years returned an initial questionnaire. The NHS breast cancer case-control study is nested within a subcohort of 32,826 women who donated blood during 1989 and 1990 and were followed until 2004 for incident disease [34, 35]. In 1989, 116,430 additional U.S. registered nurses returned an initial questionnaire (Nurses’ Health Study II [NHSII]). The NHSII breast cancer case-control study is nested within a subcohort of 29,611 women who donated blood during 1996–1999 and were followed until 2005 [36]. Medical records were used to confirm the diagnoses in women who reported a diagnosis of breast cancer on the biennial questionnaires for both NHS and NHSII. Control subjects were matched to cases based on age, menopausal status, recent hormone replacement therapy, and blood draw-specific variables (such as date and time of day). For this study, we included a total of 771 cases and 789 controls from the NHS and NHSII who have previously been genotyped as a part of a GWAS [10] and had DNA available (Additional file 1: Table S1).

The Multiethnic Cohort (MEC) is a population-based prospective cohort study (*n* = 215,251) that was initiated between 1993 and 1996 and includes subjects from various ethnic groups: African Americans and Latinos primarily from California (Greater Los Angeles area), Native Hawaiians, Japanese Americans, and European Americans primarily from Hawaii [37]. State driver’s license files were the primary sources used to identify study subjects in Hawaii and California. Additionally, in Hawaii, state voter registration files were used, and in California, Health Care Financing Administration files were used to identify additional African American study subjects. In the cohort, incident cancer cases are identified annually through cohort linkage to population-based cancer Surveillance, Epidemiology, and End Results registries in Hawaii and Los Angeles County as well as to the statewide California Cancer Registry. Blood sample collection in the MEC began in 1994 and targeted incident breast cancer cases and a random sample of study participants to serve as controls for genetic analyses. Subjects are frequency-matched on age at blood draw and on ethnicity. For this study, we included subjects who had already been genotyped as part of a GWAS [15] and had DNA available: 468 cases and 469 controls of African American ancestry, 452 cases and 458 controls of Latino ancestry, and 622 cases and 638 controls of Japanese ancestry (Additional file 1: Table S1).

### Sequencing

We selected and sequenced 12 regions because of their association with breast cancer (Additional file 2: Table S2). These regions were 2q35 (rs13387042), *TERT* (rs10069690), *MAP3K1* (rs889312), *ESR1* (rs2046210), 8q24 (rs1562430), *ZNF365* (rs10995190), *ZMIZ1* (rs704010), *FGFR2* (rs2981579), 11q13 (rs614367), *RAD51B* (rs99737), *TOX3* (rs3803662), and 19p13 (rs8170). In addition, we sequenced the *TERC* region on chromosome 3 because of its involvement in telomere length. Initial quality control (QC) was conducted on all 13 regions, but we present results for only the 12 regions associated with breast cancer here. Region boundaries were defined by nearest recombination hot spot downstream and upstream from the original GWAS signal as identified using the HapMap CEU (Utah residents with ancestry from northern and western Europe), YRI (Yoruba in Ibadan, Nigeria), JPT (Japanese in Tokyo, Japan), and CHB (Han Chinese in Beijing, China) populations. We set out to hybrid-capture and sequence a total of 5500 kb (Additional file 2: Table S2). Sequencing was conducted at The Broad Institute using an Illumina HiSeq sequencing system (Illumina, San Diego, CA, USA). Sequencing was performed using a capture method that uses biotinylated RNA “baits” to fish targets out of a “pond” of DNA fragments [38]. Agilent eArray software (Agilent Technologies, Santa Clara, CA, USA) was used to design the baits using 2× tiling frequency and a centered layout strategy, avoiding standard repeat masked regions, and allowing a maximum of 20-bp overlap with repeat masked regions. A complex pool of ultralong 200-mer oligonucleotides (“baits”) consisting of a target-specific 170-mer sequence flanked by 15 bases of a universal primer sequence on each side are synthesized in parallel on an Agilent microarray and then cleaved from the array. We then used in vitro transcription to generate a single-stranded RNA hybridization bait for fishing targets of interest out of a “pond” of randomly sheared, adaptor-ligated, and polymerase chain reaction-amplified DNA. RNA bait-DNA hybrids are “fished” out of the complex mixture by incubation with streptavidin-labeled magnetic beads and captured onto a strong magnet. After the beads are washed, the RNA bait is digested so that the only remaining nucleotide is the targeted DNA of interest. A few cycles of DNA amplification are performed at the end of the capture, and the targeted sample is then loaded onto the sequencing instrument. This method allows preparation of large amounts of bait from a single oligonucleotide array synthesis that can be tested for quality, stored in aliquots, and used repeatedly over the course of a large-scale targeted sequencing project. Within the nonrepetitive regions, we could design baits to cover 82.8 % of the sequence.

### Alignment and genotype calling

We used Burrows-Wheeler Aligner (BWA) software to align reads to the genome [39]. Genotype calling was done using GATK software with default standard filters [40, 41]. GATK takes the raw BAM files and does initial checking by correcting for possible SNP artifacts due to local realignment around indels and mark reads that were duplicately sequenced. Owing to the size of the dataset, it was not practically feasible to recalibrate the base quality scores that are provided by the sequencing machine. Therefore, we used the following filtering for SNP calling: QD <2.0, MQ <40.0, FS >60, HaplotypeScore >13.0, MQrankSum <12.5, and ReadPosRankSum <8.0. We used QD <2.0, FS >200, Read PosRankSum <20.0, and InbreedingCoeff <0.8 for indel calling. Variant calling was made in 47 different batches (about 100 samples in each batch). We randomly assigned subjects to batches after conditioning on ethnicity and case-control status to ensure full representation in each batch. All variant calls with a quality score <30 were omitted. To account for variants that were seen in only one or a few batches, we recalled all individuals in batches where the variant was not seen from missing to reference homozygous.

### Genotype and sample filtering

**Fig. 1:**
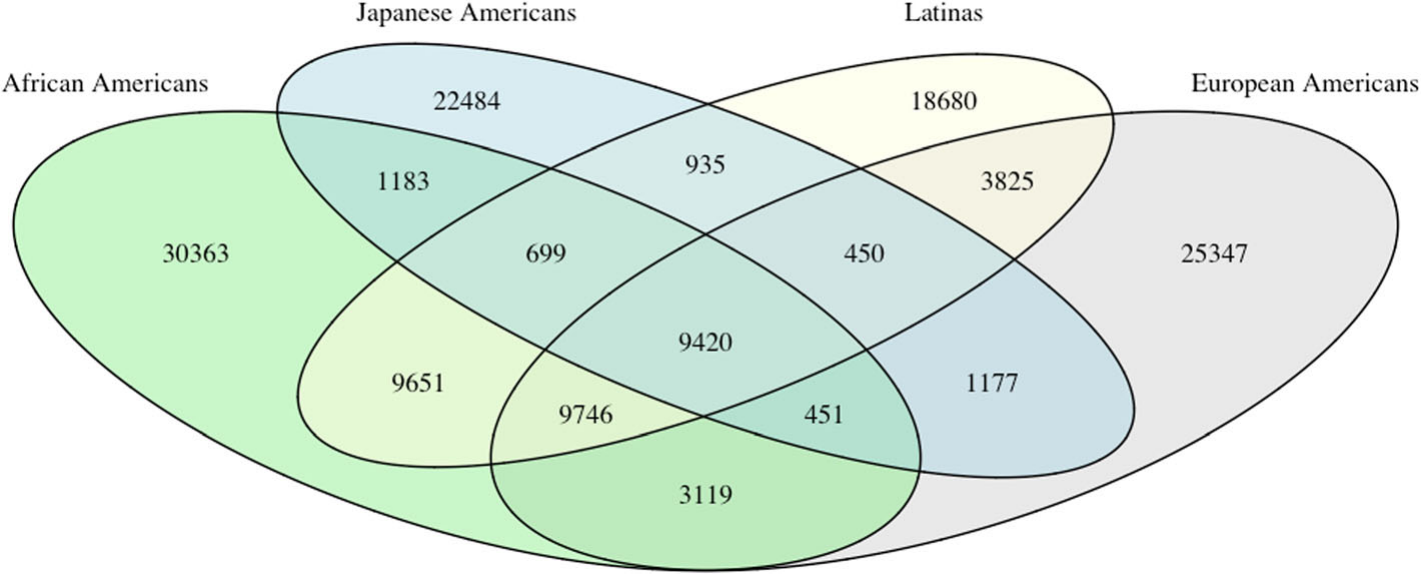
Number of single-nucleotide variants (SNVs) that passed quality control within and across ethnicities for 4611 women. The majority of variants were population-specific, and only 9420 (6.8 %) of SNVs were shared among all ethnicities

We initially observed 158,265 single-nucleotide variants (SNVs). We removed SNVs where >10 % of the samples had no reads or when the total of reads across all samples was <20,000. We then set individual genotypes to missing if the number of reads was <5 or the quality score was <10. Finally, we removed SNVs with >10 % missing or due to evidence of departure from Hardy-Weinberg equilibrium (*p* < 10^−6^) in any ancestry group. We excluded 8 samples that were unexpected pairwise duplicates, 43 samples that showed <90 % concordance with GWAS data (indicating sample mixup), 16 samples that had a call rate <90 % (Fig. 1d), 36 samples for which we did not have GWAS data, and 5 samples showing unexpected non-European ancestry. After applying these filters, there were 138,792 SNVs left for analysis.

### Baited vs. nonbaited regions

We were not able to design baits for 48.9 % of the original targeted sequences. Before QC, 32 % of the SNVs fell within nonbaited regions. Across all samples, 61.6 % of nonbaited SNVs had an average read depth >10× compared with 99.0 % of the baited SNVs, and 43.2 % of nonbaited SNVs had an average read depth >20× compared with 96.9 % of baited SNVs. After QC, 23.5 % SNVs fell in the nonbaited regions. Of those, 94.2 % had an average read depth >10× compared with 99.7 % of the baited samples, and 67.4 % of nonbaited SNVs had an average read depth >20× compared with 97.4 % of baited SNVs. Of the SNVs that were removed in the QC, 86.5 % were in nonbaited regions (Additional file 3: Figure S1).

### Coding variant annotation

Annotation of variants or assignment of a variant to a gene was implemented using GEMINI (GEnome MINIng) [42]. GEMINI is a flexible, UNIX-compatible framework for exploring genome variation that pulls information from SnpEff [43] as mapped by build 37 of the human genome. We further annotated each variant with Polymorphism Phenotyping version 2 (PolyPhen-2) scores [44], which predict the functional impact of individual variants using Variant Effect Predictor (VEP) [45]. Of the 2085 nonsynonymous coding variants that passed QC filters in our study population, we obtained PolyPhen-2 scores for 1975 (95 %). Of those, we obtained exclusive predictions for 1852 variants after excluding variants with none or unknown predictions. Lower scores correspond to less damaging qualitative values, and scores ranged from 1 to 617 (Additional file 4: Table S3). Scored variants were assigned to at least one of PolyPhen-2 qualitative prediction: *benign*; *possibly damaging*; *probably damaging*; or, in the case of insufficient data, *unknown or none*. For the rare-variant tests, we further refined the set of variants in each gene and included only nonsynonymous variants that were predicted by PolyPhen-2 to be *possibly damaging* or *probably damaging*.

### Statistical methods

We used logistic regression to assess the association between each common SNV and breast cancer risk by population as well as across populations. We conducted ethnicity-specific analysis adjusting for the top three principal components within each ethnicity to adjust for potential population stratification [46]. We combined results across ethnicities using fixed-effect meta-analysis. We adjusted for multiple testing using a modified Bonferroni correction, which allows for dependence between tests within each GWAS region by calculating a region-specific effective number of tests [47]. To assess whether regions contained multiple statistically independent risk alleles, we reran the association analysis, conditioning on the top SNP/index SNP in each region.

We also conducted an approximate Bayesian analysis to estimate the posterior probability that a given SNP is a causal variant, assuming there is only one causal SNP in the region. We estimated the posterior probability using the ratio of the likelihood from the logistic regression for a particular SNP to the sum of the likelihoods for individual SNPs in the region. The highest posterior density set is then defined as the smallest set of SNPs such that the total posterior density (summed over all SNPs in the set) is >90 %. All analyses were performed in PLINK ([48], http://pngu.mgh.harvard.edu/~purcell/plink/), R [49], and METAL [50]. We conducted additional analysis using a novel fine-mapping framework (PAINTOR) [51] that integrates external functional annotation with genetic data for prioritization of causal variants. PAINTOR jointly models multiple causal variants from all included loci simultaneously, increasing localization accuracy. We included two sets of annotations in our analysis, coding variants and variants located in DNase I hypersensitive sites (DHSs) identified in the breast tissue cell lines MCF-7, HMEC, and HMF. We ran two sets of analyses, the first assuming one causal SNP per region and the other assuming two causal SNPs per region.

We used two gene-based rare-variant tests in each ethnicity: a burden test and a sequence kernel association test (SKAT) [52]. Each association test was performed separately by ethnicity and adjusted for the first three ethnicity-specific principal components. To combine evidence across ethnicities, we applied two meta-analytic techniques: inverse-variance (BURDEN)-weighted fixed-effect meta-analysis [53] and meta-analysis of SKAT assuming the effect of each variant is homogeneous, regardless of ethnicity (Hom-Meta-SKAT) [54]. Each technique uses a distinct approach: a mean-based approach for fixed-effect meta-analysis and a variance-based approach for Hom-Meta-SKAT.

## Results

We sequenced 937 women of African American ancestry, 1256 women of Japanese American ancestry, 907 women of Hispanic ancestry, and 1511 women of European ancestry (Additional file 1: Table S1). Subjects were participants in NHS [34], NHSII [36], and MEC [37]. In total, we sequenced 2288 breast cancer cases and 2323 controls. We were not able to capture a total of 2740 kb (49.8 %) originally targeted, primarily because of repetitive sequence content. The median proportion of captured regions with coverage >20× was higher than 93 % across all regions (range 93.8–99.9 %).

After quality control of the 12 regions (see the Methods section above), we obtained genotype data on 137,530 SNVs. Of those, 34,532 (25.11 %) were located in intergenic regions, 62,427 (45.39 %) were intronic, 1306 (0.95 %) were synonymous, and 1983 (1.44 %) were nonsynonymous. The rest (27 %) were located upstream/downstream genes, 3′ and 5′ untranslated regions, and in coding regions (e.g., nonsense variants) (Additional file 5: Table S4). On average, each region contained 6.3 genes (range 1–26), and the median number of SNVs by region was 8401 (range 1833–23,757). We observed an abundance of rare SNVs, with 119,980 SNVs (87.2 %) having a minor allele frequency (MAF) <0.005; of these, 64,747 SNVs (47.1 %) were private mutations (54 variants were homozygous in one carrier). The number of polymorphic variants ranged from 36,799 in Japanese Americans to 64,632 in African Americans. Most variants were population-specific: 70 % of all variants were observed in only one ethnicity (Fig. 1), emphasizing the genetic diversity across populations. In contrast, a total of 9420 (6.8 %) SNVs were shared across all ethnicities. In general, Japanese Americans had the largest proportion of SNVs not shared with others (61 %), whereas Latinas had the smallest proportion of population-specific SNVs (35 %).

The majority of observed SNVs were novel: Only 27.3 % were observed in the 1000 Genomes Project [55] (Additional file 6: Figure S2, Additional file 7: Figure S3, and Additional file 8: Figure S4). Due in part to the low read depth in uncaptured repetitive sequences, 42,105 (47.4 %) of the SNVs present in the 1000 Genomes Project for these regions were not observed in the targeted sequencing data (Additional file 9: Figure S5). However, we observed 26,205 (73.4 %) of the 35,714 1000 Genomes Project SNVs that were located in captured regions in our targeted sequencing data, suggesting that the majority of 1000 Genomes Project SNVs that were not observed in our targeted sequencing data were located within regions not captured by our sequencing technology.

We first conducted individual SNP analysis of common variants in at least one ancestry (MAF >0.005, *n* = 27,380). After Bonferroni correction, we did not observe any significant associations in ethnicity-specific analyses (data not shown) or across all ethnicities (Additional file 10: Table S5). Given the strong correlation between SNPs in these regions, we also applied a more relaxed *p* value threshold, adjusting for number of effective tests [47]. Using this approach, we observed four SNPs that remained statistically significant after correcting for multiple testing (*p* < 4.59 × 10^−6^), all in the 11q13 region (rs61041893, rs7123796, rs597587, rs644376). These SNPs are all within 13 kb of each other, and SNPs rs7123796, rs597587, and rs644376 are all in strong LD with each other (*r* ^2^ > 0.74), whereas rs61441893 show only moderate correlation with the other SNPs (*r* ^2^ = 0.31–0.39) with the others. Interestingly, these SNPs are not correlated with the GWAS index SNP rs614367 (*r* ^2^ < 0.01) and approximately 40 kb away from the nearest gene (*CCND1*). The strongest association was observed for rs61041893 (OR 0.63, 95 % CI 0.52–0.76, *p* = 2.15 × 10^−6^). Of note, this SNP was evaluated only in African Americans and Hispanics because it was not observed in Japanese Americans and was very rare (MAF = 0.002) in European Americans. Of the 12 GWAS index SNPs previously reported in the literature, we replicated 6 across all populations using an unadjusted *p* value threshold of 0.05 (Table 1).

**Table 1:** Breast cancer association results

Region	Index SNP (locus)	Chromosome	Length (Mbp)	SNV MAF ≤0.005	SNV MAF >0.005	High-impact SNVs^b^	*p* value (index SNP)	*p* value (top SNP)	Conditional analysis on top SNP *p* value (top SNP)	Conditional analysis on index SNP *p* value (top SNP)
1	rs13387042 (*2q35*)	2	0.122	3469	674	0	0.00016	2.68E-05 (rs6721996)	0.0070 (rs116670542)	0.0066 (rs116670542)
2	rs10069690 (*TERT*)	5	0.046	1681	167	0	0.0044	0.0044 (rs10069690)	0.025 (rs34768248)	0.025 (rs34768248)
3	rs889312 (*MAP3K1*)	5	0.308	6663	986	4	0.098	0.00035 (rs111944656)	0.0022 (rs79128470)	0.0021 (rs111944656)
4	rs2046210^a^ (*ESR1*)	6	0.243	4209	727	10	0.015	0.00023 (rs9383938)	0.0074 (rs80347946)	0.0077 (rs80347946)
5	rs1562430 (*8q24*)	8	0.973	20,730	3260	5	0.18	9.87E-05 (rs112613843)	0.00020 (rs4871810)	0.00019 (rs4871841)
6	rs10995190 (*ZNF365*)	10	0.876	7635	1056	1	0.76	2.7E-05 (rs12570941)	0.0085 (rs73282644)	5.39E-05 (rs12570941)
7	rs704010 (*ZMIZ1*)	10	0.398	13,622	1822	4	0.019	0.00025 (Chr10-81107117)	0.00076 (rs117770051)	0.00019 (Chr10-81107117)
8	rs2981579 (*FGFR2*)	10	0.473	20,081	3033	15	0.00046	4.42E-05 (rs10736303)	0.00020 (rs192776427)	0.00024 (rs192776427)
9	rs614367 (*11q13*)	11	0.259	6743	1010	5	0.75	2.15E-06 (rs61041893)	0.00032 (rs11823311)	5.47E-05 (rs598003)
10	rs999737 (*RAD51B*)	14	0.815	15,992	2025	8	0.067	0.00050 (rs76904544)	0.00086 (rs113627141)	0.00074 (rs113627141)
11	rs3803662 (*TOX3*)	16	0.269	7337	910	0	0.036	0.00013 (rs12922061)	0.0013 (rs8048809)	0.0013 (rs4784227)
12	rs8170 (*MERIT40*)	19	0.712	13,080	1880	31	0.67	0.0035 (rs62126223)	0.0069 (rs117673644)	0.0035 (rs62126223)

*MAF* Minor allele frequency, *SNP* Single-nucleotide polymorphism, *SNV* Single-nucleotide variant

The results shown are derived from genome-wide association study index single-nucleotide polymorphisms and best associated single-nucleotide variant in sequenced regions spanning 12 breast cancer genome-wide association study loci. Results are also shown for conditional analysis adjusted for either best associated (“top”) SNP or the original index genome-wide association study SNP

^a^SNP was filtered in quality control, *p* value for rs12662670

^b^As defined by SnpEff [43]

**Fig. 2:**
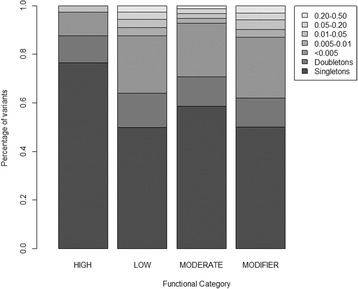
Minor allele frequency distribution of observed single-nucleotide variants (SNVs). SNVs are categorized by functional impact (high, moderate, low, or modifier) as predicted by the SnpEff algorithm [43]. High (*n* = 81) is defined as disruptive impact SNVs, moderate (*n* = 1983) as nonsynonymous coding SNVs, low (*n* = 1374) as synonymous coding SNVs, and modifier (*n* = 134,092) as noncoding SNVs

We used SnpEff [43] to annotate and predict SNV effects. We observed 81 SNVs that had a predicted disruptive impact (e.g., splice site donators/acceptors, loss of start/stop codon, gained stop codon, frame shift variants), 1983 nonsynonymous coding SNVs, 1374 synonymous coding SNVs, and 134,092 noncoding SNVs (Fig. 2, Additional file 5: Table S4). Two SNVs with predicted disruptive function had a MAF >0.005 in the full dataset. SNP rs79619171 in the *FGFR2* region is a splice site donor in the *TACC2* gene; it was observed only in the Japanese American samples (MAF 0.08) and was moderately associated with breast cancer (OR 1.47, 95 % CI 1.09–1.98, *p* = 0.01). SNP rs55670604 is a splice site acceptor in the *RAD51L1* gene; it was observed in Latinas (MAF 0.03), African Americans (MAF 0.02), and European Americans (MAF 0.08), but it was not associated with breast cancer in any population (data not shown) or across all ethnicities (OR 1.00, *p* = 0.99).

To identify multiple independent signals, we reran the association analysis, conditioning on the top SNP in each region. We observed no new significant associations after correcting for multiple testing (all *p* > 10^−4^) (Table 1). We also assessed if there was evidence of additional signals beyond the index SNP in these regions by conditioning on the original GWAS index SNP (Table 1). For the *ZNF365* and 11q13 regions, we observed evidence of association signals beyond the index signal (*p* < 10^−4^), in agreement with previous studies [5, 56, 57]. We also conducted an approximate Bayesian analysis [58] to estimate the posterior probability that a given SNP is a causal variant, assuming there is only one causal SNP in the region (Additional file 11: Figure S6). The highest posterior probability set (PPS) is then defined as the smallest set of SNPs such that the total posterior density (summed over all SNPs in the set) is >90 % and can help guide the selection of candidate SNPs for further downstream functional and bioinformatic analyses. The number of SNPs included in the highest posterior density set varied widely, between 11 (11q13) and 2954 (8q24), in analysis across all ethnicities (Table 2). For two regions (2q35 and 11q13), <5 % of the original SNVs were needed to obtain a 90 % PPS. Population-specific analysis showed similar results (data not shown). We conducted additional fine-mapping analyses using a novel fine-mapping framework (PAINTOR) [51] that integrates external functional annotation with genetic data and calculates SNV-specific posterior probabilities for causality. We included two sets of annotations in our analysis: coding variants and variants located in DHSs identified in the breast tissue cell lines MCF-7, HMEC, and HMF [59]. We limited our analysis to SNPs with an allele frequency >0.01. In total, we included 13,373 SNPs, and of those, 104 (0.8 %) were coding, 371 (2.8 %) were located in breast tissue DHSs, and one SNP was both coding and located in a DHS. We ran two sets of analyses, the first assuming one causal SNP per region and the other assuming two causal SNPs per region. Overall, the results from either of these analyses did not qualitatively differ from the Bayesian analysis without incorporating functional annotation data (Additional file 12: Table S6). However, for the 11q13 region, we noticed that while both the Bayesian approach and PAINTOR assuming one causal variant predicted that rs61041893 had the highest posterior probability (0.22 using both approaches), this SNP had a posterior probability of only 2.2 × 10^−5^ when we ran PAINTOR assuming two causal variants. Instead, rs12279395 and rs11823311 both had high posterior probabilities (>0.99) of being causal. These two variants were located 74 kb and 75 kb apart from rs61041893, respectively, and 150 kb apart from each other, They were both nominally associated with breast cancer risk (*p* < 0.0005). SNP rs12279395 is a nonsynonymous SNP located in the *ORAOV1* gene, whereas rs11823311 is located in an intergenic region. Interestingly, these three variants (rs12279395, rs11823311, and rs61041893) all show regulatory properties in breast tissue in ENCODE [59] as defined by HaploReg [60].

**Table 2:** Results of posterior probability analysis

Region	Index SNP (locus)	Chromosome	SNPs in PPS (proportion)	Proportion of SNPs in PPS (all SNPs) according to functional annotation^a^			
				High impact	Moderate impact	Low impact	Modifier impact
1	rs13387042 (*2q35*)	2	42 (0.04)	0 (0)	0 (0)	0 (0)	1 (1.00)
2	rs10069690 (*TERT*)	5	224 (0.74)	0 (0)	0.009 (0.01)	0.027 (0.023)	0.964 (0.967)
3	rs889312 (*MAP3K1*)	5	802 (0.58)	0 (0)	0.005 (0.005)	0.012 (0.012)	0.983 (0.983)
4	rs2046210 (*ESR1*)	6	455 (0.44)	0 (0)	0.026 (0.019)	0.009 (0.01)	0.965 (0.971)
5	rs1562430 (*8q24*)	8	2954 (0.59)	0 (0)	0.001 (0.003)	0.001 (0.001)	0.997 (0.996)
6	rs10995190 (*ZNF365*)	10	716 (0.41)	0 (0)	0.007 (0.006)	0 (0.003)	0.993 (0.992)
7	rs704010 (*ZMIZ1*)	10	2108 (0.72)	0 (0)	0.001 (0.001)	0.007 (0.005)	0.992 (0.994)
8	rs2981579 (*FGFR2*)	10	2827 (0.59)	0.0004 (0.0002)	0.01 (0.011)	0.007 (0.008)	0.982 (0.981)
9	rs614367 (*11q13*)	11	11 (0.007)	0 (0)	0 (0.007)	0 (0.002)	1 (0.990)
10	rs999737 (*RAD51B*)	14	2451 (0.77)	0 (0.0003)	0.004 (0.005)	0.003 (0.003)	0.993 (0.991)
11	rs3803662 (*TOX3*)	16	850 (0.55)	0 (0)	0.001 (0.003)	0.007 (0.006)	0.992 (0.991)
12	rs8170 (*MERIT40*)	19	2274 (0.79)	0 (0)	0.035 (0.035)	0.049 (0.047)	0.916 (0.917)

*PPS* Posterior probability set, *SNP* Single-nucleotide polymorphism

Results are shown for PPSs and proportions of PPS SNPs by functional annotations compared with the overall distribution of annotations by region

^a^As defined by SnpEff [43]

Three of the sequenced regions have shown a stronger association with estrogen receptor negative (ER^−^) breast cancer [4, 9, 61], which is a more aggressive subtype of breast cancer. A total of 393 breast cancer cases in our data were ER^−^. Recognizing the limited power of our study, we reran the analysis with ER^−^ breast cancer as the outcome. No SNP was significantly associated with ER^−^ breast cancer after our analysis was adjusted for number of tests (Additional file 13: Table S7). The strongest associated SNP was rs112613843 on 8q24 (*p* = 0.0002). However, this SNP was observed only in African Americans. In population-specific analysis, the GWAS index SNP rs10069690 in the *TERT* gene was significantly associated with ER^−^ breast cancer in African Americans (*p* = 1.11 × 10^−6^) but in no other population (all *p* > 0.3).

**Fig. 3:**
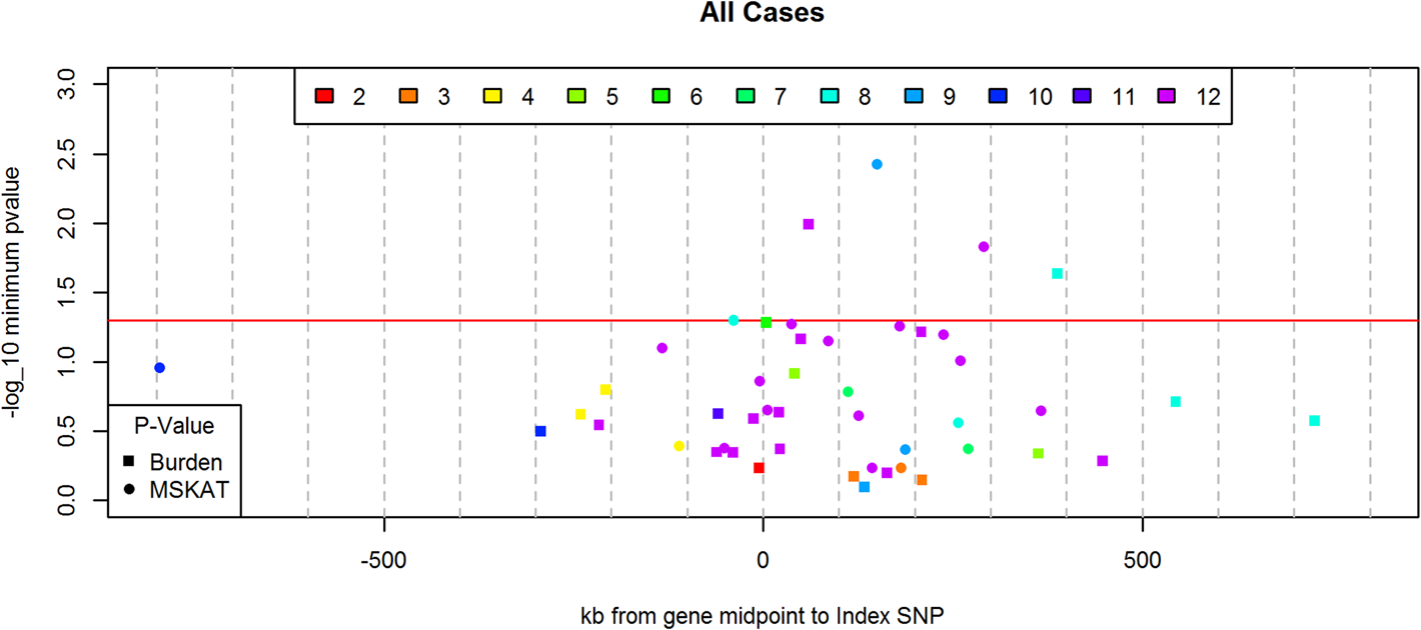
Gene-based rare-variant association for overall breast cancer by distance from index genome-wide association study (GWAS) single-nucleotide polymorphism (SNP). *y*-Axis displays log_10_ gene association *p* values. *Horizontal red line* represents alpha = 0.05. Only the lower of two *p* values is plotted. For round points, the sequence kernel association test had the lower *p* value, and for square points, the burden test had a lower *p* value. Points are color-coded for the 12 breast cancer GWAS-identified index SNPs on 9 chromosomes. Region legend: 2 = *TERT*, 3 = *MAP3K1*, 4 = *ESR1*, 5 = 8q24, 6 = *ZNF365*, 7 = *ZMIZ1*, 8 = *FGFR2*, 9 = 11q13, 10 = *RAD51B*, 11 = *TOX3* and 12 = 19p13

Of the 12 sequenced regions, 11 contained coding regions with rare (MAF <0.005), nonsynonymous variation. For each of the 47 genes in these 11 regions, we performed 2 aggregate tests of association between breast cancer risk (overall and by ER status) and all nonsynonymous rare variants (a burden test and SKAT [52]); we also repeated these tests restricting the analysis to nonsynonymous rare alleles predicted to be damaging by PolyPhen-2 [44]. Tests were conducted stratified by ethnicity (Additional file 14: Table S8) and then meta-analyzed across ethnicities (Table 3). After applying the Bonferroni correction for the number of genes tested, we did not observe any significant findings using either SKAT or the burden test: The smallest *p* value was 0.004 (SKAT) for overall breast cancer when we included all nonsynonymous rare variants in *ORAOV1* on chromosome 11q13 (Table 3, Fig. 3). Of note, rs12279395, which was highlighted by the PAINTOR analysis in the two-causal model, is a common nonsynonymous variant (MAF 0.12) in *ORAOV1*, providing additional support that *ORAOAV1* is important in breast cancer development.

**Table 3:** Nominally significant rare-variant tests for breast cancer overall and by estrogen receptor status, across all ethnicities

Outcome	Gene	Chromosome: index SNP	Number of variants	CP	Significant test(s)	*p* value	OR (95 % CI)
Breast cancer overall	*ORAOV1*	Chr11: rs614367	43	4.80 %	SKAT	0.004	0.8 (0.6–1.1)
	*GTPBP3*	Chr19: rs8170	48	3.50 %	SKAT	0.01	1.3 (0.9–1.8)
	*GLT25D1*	Chr19: rs8170	55	2.50 %	SKAT	0.015	0.9 (0.7–1.4)
	*DDA1 - p2*	Chr19: rs8170	1	<1.0 %	SKAT	0.046	2.0 (0.9–4.1)
	*NSMCE4A*	Chr10: rs2981579	17	0.70 %	BURDEN	0.023	0.4 (0.2–0.9)
	*ANO8 - p2*	Chr19: rs8170	8	<1.7 %	BURDEN	0.034	5.3 (1.1–24.2)
ER^+^	*GTPBP3*	Chr19: rs8170	48	3.50 %	SKAT	0.007	1.4 (1.0–2.1)
	*TMEM221 - p2*	Chr19: rs8170	5	<2.1 %	SKAT	0.013	0.4 (0.2–1.0)
	*GLT25D1*	Chr19: rs8170	55	2.50 %	SKAT	0.018	0.8 (0.5–1.3)
	*ZNF365 - p2*	Chr10: rs10995190	8	<2.0 %	SKAT, BURDEN	0.019	2.5 (1.0–5.9)
	*ORAOV1*	Chr11: rs614367	43	4.80 %	SKAT	0.024	0.8 (0.6–1.1)
	*TMEM221*	Chr19: rs8170	24	2.10 %	SKAT	0.028	0.6 (0.4–1.1)
	*ZFYVE26*	Chr14: rs999737	126	5.70 %	SKAT	0.041	1.2 (0.9–1.5)
	*MAP1S - p2*	Chr19: rs8170	22	<4.9 %	SKAT	0.045	0.9 (0.6–1.5)
	*GLT25D1 - p2*	Chr19: rs8170	15	<2.5 %	SKAT	0.049	0.9 (0.4–1.9)
	*ZNF365*	Chr10: rs10995190	38	2.00 %	BURDEN	0.013	1.8 (1.1–2.9)
	*PLVAP*	Chr19: rs8170	31	1.80 %	BURDEN	0.016	1.8 (1.1–3.0)
	*FGFR2*	Chr10: rs2981579	41	1.50 %	BURDEN	0.016	2.0 (1.1–3.4)
	*C6orf211*	Chr6: rs2046210	34	2.70 %	BURDEN	0.044	0.6 (0.4–1.0)
ER^−^	*ORAOV1*	Chr11: rs614367	43	4.80 %	SKAT	0.028	0.9 (0.5–1.4)
	*ANO8 - p2*	Chr19: rs8170	8	<1.7 %	SKAT	0.028	4.4 (0.3–74.5)
	*FAM129C*	Chr19: rs8170	62	6.80 %	SKAT	0.035	0.9 (0.6–1.3)
	*BABAM1*	Chr19: rs8170	24	1.40 %	SKAT	0.041	2.1 (0.9–5.1)
	*ORAOV1 - p2*	Chr11: rs614367	2	<4.8 %	SKAT	0.042	3.3E + 06 (0.0– > 1E50)
	*UNC13A*	Chr19: rs8170	69	3.70 %	SKAT	0.045	1.5 (0.9–2.5)
	*ZFYVE26*	Chr14: rs999737	126	5.70 %	SKAT	0.049	1.5 (1.0–2.3)
	*USE1*	Chr19: rs8170	14	0.40 %	BURDEN, SKAT	0.022	7.2 (1.3–38.8)
	*ABHD8*	Chr19: rs8170	19	1.30 %	BURDEN	0.026	2.4 (1.1–5.0)
	*TMEM221*	Chr19: rs8170	24	2.10 %	BURDEN	0.029	2.1 (1.1–3.9)
	*ZFYVE26 - p2*	Chr14: rs999737	29	<5.7 %	BURDEN	0.031	2.5 (1.1–5.7)

*Abbreviations: SNP* Single-nucleotide polymorphism, *p2* Subset of variants within gene predicted to be potentially or possibly damaging by Polymorphism Phenotyping version 2, *CP* Carrier proportion or proportion of subjects who carry at least one rare, nonsynonymous variant in gene, *BURDEN* Inverse-variance-weighted burden test, SKAT Meta-analysis of sequence kernel association test assuming the effect of each variant is homogeneous, regardless of ethnicity

*Underlining* = lowest *p* value if more than one significant test

## Discussion

In this study, we sequenced 12 genetic regions that have been found to be associated with breast cancer in 2288 breast cancer cases and 2323 controls. We found no strong evidence for a single causal allele in any of the regions. It is likely that the lack of strong signals in our data is due to the inadequate sample size resulting in a low signal-to-noise ratio. The initial GWASs identifying these loci were larger than our study population for many of these regions. It has been shown that fine-mapping studies that use multiple ethnic populations and leverage the genetic variability across populations have greater ability to localize causal variants [28]. Although we included four different ethnicities in this study, our total sample size of 4611 subjects was most likely too small for pinpointing causal variants with high probability. Further, subsequent population-specific efforts have shown that not all regions are associated with breast cancer across ethnicities. A recent study of 3016 cases and 2745 controls of African American ancestry replicated only 4 (2q35, *TERT, FGFR2*, and *MERIT40*) of the 12 regions investigated here at *p* < 0.05 [30]. A study of up to 15,130 cases and 14,584 controls of East Asian descent replicated 7 (2q35, *MAP3K1*, *ESR1*, *ZMIZ1*, *FGFR2*, 11q13, and *TOX3*) of the 12 regions at a significance level of 0.05 (although strong evidence has been found for the rs10822013 SNP located in the *ZNF365* region). In addition, the *TERT* region was associated with ER^−^ breast cancer [62]. In Latinas, 4 (*MAP3K1*, *ZMIZ1*, *FGFR2*, and *TOX3*) of the 12 regions have been associated with breast cancer in 1497 cases and 3213 controls [27]. However, when taking tumor subtypes into account, associations were also observed for ER^−^ breast cancer (*MERIT40*) and 2q35 and *ZNF365* (ER^+^ breast cancer).

Another weakness of our study was the incomplete capture of our targeted regions. The capture method we used was able to capture only 50.1 % of the original targeted regions (range 38.8–79.1 %). Nevertheless, we discovered a large proportion of novel variants not observed in the 1000 Genomes Project, illustrating the importance of sequencing depth as well as large, diverse populations to obtain a comprehensive catalogue of the genetic variation within a specific region. Despite the large number of novel and low-frequency variants, we did not detect a significant association between rare, nonsynonymous variation and breast cancer risk. Of note, the vast majority (>98 %) of the variants sequenced in our study were outside coding regions, and one region, 2q35, did not contain any rare, nonsynonymous variants.

Earlier breast cancer fine-mapping studies [30, 56, 63–65] identified multiple candidates for causal variants, but it remains a challenge to determine the evidence required to confidently declare a variant causal. In contrast to previous studies, this is the first study, to our knowledge, to use sequence data rather than genotyped and imputed data, greatly improving genomic coverage. We attempted to identify secondary signals by running conditional analyses as well as using Bayesian approaches to identify the best candidate(s) for causal variants. For two of the regions, 2q35 and 11q13, the Bayesian analysis allowed us to create 90 % PPSs including <5 % of the original SNVs, greatly reducing the number of potential candidate causal SNVs. We also incorporated functional annotations with the goal of upweighting SNVs that were of functional importance. We included coding and breast-specific DHS as our two annotations, but none of these annotations showed evidence of being enriched for causal SNVs. It is possible that our lack of findings for these annotations is due either to limited power in our analysis or to these annotations not truly being enriched for causal breast cancer SNVs. Generation of large-scale databases including functional annotations throughout the genome is a constantly evolving area, and, as more data become available, annotations such as those used here can readily be updated and expanded.

For the 2q35 region, our results agree with those of a previous fine-mapping study [63] of the same region. On the basis of data from 46,451 cases and 42,599 controls of European ancestry and 6269 cases and 6624 controls of Asian ancestry in the Breast Cancer Association Consortium (BCAC), the investigators found evidence that one of two highly correlated SNPs (rs4442975 and rs6721996) is likely to explain the association signal observed in this region. This is in agreement with our results where we found that rs6721996 (*p* = 2.68 × 10^−5^, posterior probability 0.29) and the strongly correlated rs13412666 (*p* = 2.98 × 10^−5^, posterior probability 0.25) showed the strongest association in our data. SNP rs6721996 is also strongly correlated (*r* ^2^ = 0.97) with the original GWAS SNP rs13387042. Our results, together with the BCAC results, argue that the breast cancer signal from 2q35 can be explained with only a few SNPs.

In another fine-mapping study [66] by the BCAC, the authors found evidence for three independent signals in the 5q11.2 (*MAP3K1*) region. After adjustment for multiple testing, we did not identify any significant associations in this region; however, our top finding (rs111944656, *p* = 0.0004) is correlated (*r* ^2^ = 0.63) with one of the top signals in their study (rs113317823). The BCAC also explored the *FGFR2* region at 10q26 [64] and found three independent signals. Our top SNP, rs10736303 (*p* = 4.42 × 10^−5^), is strongly correlated with two of their signals (rs2981578 [*r* ^2^ = 0.94] and rs2912779 [*r* ^2^ = 0.79]). The 11q13 region was the only region where the results dramatically changed if we assumed two causal variants rather than one in our PAINTOR analysis. The initial top SNP rs61041893 lies between rs12279375 and rs11823311, and all three SNPs are in low to modest LD in our data (*r* ^2^ = 0.01–0.30). In a previous fine-mapping study [57] of this region, the BCAC investigators identified three independent regions. Unfortunately, we did not capture their top variants in our data, making it difficult to directly compare the results. However, on the basis of our and their results, it seems likely that multiple independent breast cancer associations exist in this region.

We used two approaches – SKAT and a burden test – to assess if rare genetic variations in these regions were associated with breast cancer. We limited our analysis to nonsynonymous SNPs and conducted additional analysis including only nonsynonymous variants predicted to be damaging. After adjusting for the number of tests conducted, we did not observe any evidence that rare variations in these regions affect breast cancer risk. Despite some limitations, our study population is relatively large for a sequencing study and incorporates multiple ethnicities. Mensah-Ablorh et al. [67] found that multiethnic studies with genetically diverse subjects were better powered than some single-ethnicity study populations. However, in this case, the power gain derived from including multiple ethnicities was not large enough to overcome the small number of cases and controls (<5000) included in the analysis. For genes where a given population did not carry much rare variation, multiethnic meta-analysis allowed detection of gene-based rare-variant associations that may have been missed in a monoethnic study. Indeed, sufficiently large sample sizes that incorporate ancestral populations with greater genetic diversity and that target regions appropriate to the phenotype under study are critical for effective fine-mapping.

This study makes multiple important contributions to the research field. First, the inclusion of multiple ethnicities allowed us to explore the diversity of genetic variation in these regions and highlight the importance of conducting well-powered studies within multiple ethnicities. Further, we show evidence that sequencing, as compared with genotyping, variants identified through an existing database (e.g., the 1000 Genomes Project) results in identification of many additional rare variants. On the basis of our results, we show that, for breast cancer specifically, there are no rare variants of very large effect lingering at known GWAS loci.

We make the following recommendations for future study design. Future fine-mapping studies should conduct more comprehensive sequencing (whole genome rather than capture) to fully capture the genetic variation in a region. Further, we argue that follow-up studies require even larger and carefully selected study populations than the initial GWAS.

## Conclusions

We report the first large-scale follow-up of breast cancer susceptibility loci using sequencing. We did not find any strong evidence for a single causal variant in any of the regions; however, we were able to narrow the number of potential causal SNVs in two regions (2q35 and 11q13). In addition, we did not find evidence that rare genetic variation in these regions is associated with breast cancer risk. This study illustrates some of the challenges faced in fine-mapping studies in the post-GWAS era.

## Additional files

Additional file 1: Table S1. Characteristics of breast cancer cases and controls included in this study. (DOCX 62 kb)
Additional file 2: Table S2. Selected and sequenced regions. All regions have previously been identified as breast cancer GWAS susceptibility regions in European ancestry populations. (DOCX 60 kb)
Additional file 3: Figure S1. Diagram describing the initial QC pipeline for the 12 breast cancer GWAS regions as well as the *TERC* region that was later excluded from analysis because it has not been reported to be associated with breast cancer. (DOCX 135 kb)
Additional file 4: Table S3. Counts and percentages of predictions and PolyPhen-2 scores for nonsynonymous SNVs across all 12 regions. (DOCX 46 kb)
Additional file 5: Table S4. Observed SNVs classified by their impact on gene function as predicted by SnpEff [43]. (DOCX 66 kb)
Additional file 6: Figure S2. MAF distributions of the 137,530 observed SNVs in the 12 breast cancer GWAS regions across 4 ethnicities. (DOCX 51 kb)
Additional file 7: Figure S3. MAF distribution for SNVs observed in both this study and the 1000 Genomes Project. (DOCX 50 kb)
Additional file 8: Figure S4. MAF distribution for SNVs observed in this study but not in the 1000 Genomes Project. SNVs observed in this study but not in the 1000 Genomes Project were almost exclusively very rare (MAF <0.005), and only 0.4 % (390 variants) of SNVs not observed in the 1000 Genomes Project had a MAF >0.005 compared with 46 % (17,160) of variants observed in the 1000 Genomes Project. (DOCX 54 kb)
Additional file 9: Figure S5. MAF distribution for SNVs in the 1000 Genomes Project but not in this study. (DOCX 49 kb)
Additional file 10: Table S5. Overall and ethnicity-specific associations between SNVs with MAF >0.005 and breast cancer risk in 2288 cases and 2323 controls across four ethnicities. *EA* European Americans, *AA* African Americans, *LA* Latinas, *JA* Japanese Americans, *MAF* Minor allele frequency. (XLSX 6660 kb)
Additional file 11: Figure S6. Regional association plots (*black, left panel*) and posterior probability plots (*gray, right panel*) by region. Results are based on meta-analysis across all ethnicities. The original index GWAS SNP is highlighted in *red*. (DOCX 200 kb)
Additional file 12: Table S6. SNV-specific posterior probabilities from Bayesian and PAINTOR analyses. (XLSX 1538 kb)
Additional file 13: Table S7. Associations between SNVs with MAF >0.005 and ER− breast cancer risk in 393 cases and 2323 controls, across and by ethnicity. *EA* European Americans, *AA* African Americans, *LA* Latinas, *JA* Japanese Americans, *MAF* Minor allele frequency. (XLSX 5049 kb)
Additional file 14: Table S8. Gene-based rare-variant associations by ethnicity (*p* < 0.02 in at least one ethnicity), overall cancer, and by ER status. (XLSX 51 kb)

## Abbreviations

AA: African Americans
BCAC: Breast Cancer Association Consortium
BWA: Burrows-Wheeler Aligner
CEU: Utah residents with ancestry from northern and western Europe
CHB: Han Chinese in Beijing, China
CP: Carrier proportion
DHS: DNase I hypersensitive site
EA: European Americans
ER: Estrogen receptor
FS: FisherStrand
GEMINI: GEnome MINIng
GWAS: Genome-wide association study
Hom-Meta-SKAT: Meta-analysis of sequence kernel association test assuming the effect of each variant is homogeneous, regardless of ethnicity
JA: Japanese Americans
YRI: Yoruba in Ibadan, Nigeria
JPT: Japanese in Tokyo, Japan
LA: Latinas
LD: Linkage disequilibrium
MAF: Minor allele frequency
MEC: Multiethnic Cohort
MQ: RMS Mapping Quality
NHS: Nurses’ Health Study
NHSII: Nurses’ Health Study II
PolyPhen-2: Polymorphism Phenotyping version 2
PPS: Posterior probability set
QC: Quality control
QD: Qual By Depth
SEER: Surveillance, Epidemiology, and End Results
SKAT: Sequence kernel association test
SNP: Single-nucleotide polymorphism
SNV: Single-nucleotide variant
VEP: Variant Effect Predictor
QD: Qual By Depth
FS: Fisher Strand
MQ: RMS MappingQuality
