# Supplementary material for: Deep targeted sequencing of 12 breast cancer susceptibility regions in 4611 women across four different ethnicities
Source: Breast Cancer Res. 2016 Nov 5;18:109. doi: 10.1186/s13058-016-0772-7 (PMC5097387; doi:10.1186/s13058-016-0772-7)
Supplement: Additional file 1: Table S1. — Characteristics of breast cancer cases and controls included in this study. (DOCX 62 kb) [file 13058_2016_772_MOESM1_ESM.docx]

**Table S1**. Characteristics of Breast Cancer Cases and Controls included in this study

|  |  | **Controls** | **All Cases** | **ER+ Cases** | **ER- Cases** | **All Subjects** |
| --- | --- | --- | --- | --- | --- | --- |
|  |  | N (%) | N (%) | N (%) | N (%) | N (%) |
| **Study** |  |  |  |  |  |  |
|  | MEC | 1,562 (67%) | 1,538 (67%) | 908 (66%) | 272 (69%) | 3,100 (67%) |
|  | NHS | 761 (33%) | 467 (20%) | 315 (23%) | 80 (20%) | 1,228 (27%) |
|  | NHSII | 0 (0%) | 283 (12%) | 143 (10%) | 41 (10%) | 283 (6%) |
| **Ethnicity** |  |  |  |  |  |  |
|  | African American | 469 (20%) | 468 (20%) | 273 (20%) | 117 (30%) | 937 (20%) |
|  | European American | 761 (33%) | 750 (33%) | 458 (34%) | 121 (31%) | 1,511 (33%) |
|  | Japanese American | 637 (27%) | 619 (27%) | 352 (26%) | 57 (15%) | 1,256 (27%) |
|  | Latina American | 456 (20%) | 451 (20%) | 283 (21%) | 98 (25%) | 907 (20%) |
|  | **Total** | **2,323** | **2,288** | **1,366** | **393** | **4,611** |
