# Supplementary material for: Deep targeted sequencing of 12 breast cancer susceptibility regions in 4611 women across four different ethnicities
Source: Breast Cancer Res. 2016 Nov 5;18:109. doi: 10.1186/s13058-016-0772-7 (PMC5097387; doi:10.1186/s13058-016-0772-7)
Supplement: Additional file 2: Table S2. — Selected and sequenced regions. All regions have previously been identified as breast cancer GWAS susceptibility regions in European ancestry populations. (DOCX 60 kb) [file 13058_2016_772_MOESM2_ESM.docx]

**Table S2.** Selected and sequenced regions. All regions have previously been identified as breast cancer GWAS susceptibility regions in European ancestry populations

| **Region** | **Index SNP** | **Chromosome** | **Start** | **End** |
| --- | --- | --- | --- | --- |
| 1 | rs13387042 | 2 | 217856966 | 217978765 |
| 2 | 10069690 | 5 | 1251287 | 1297162 |
| 3 | rs889312 | 5 | 55990143 | 56297954 |
| 4 | rs2046210 | 6 | 151765174 | 152007889 |
| 5 | rs1562430 | 8 | 127830818 | 128803680 |
| 6 | rs10995190 | 10 | 64083915 | 64481771 |
| 7 | rs704010 | 10 | 80653082 | 81126285 |
| 8 | rs2981579 | 10 | 123187843 | 124064057 |
| 9 | rs614367 | 11 | 69281227 | 69540165 |
| 10 | rs999737 | 14 | 68236495 | 69051900 |
| 11 | rs3803662 | 16 | 52421917 | 52690887 |
| 12 | rs8170 | 19 | 17136590 | 17849008 |
