# Supplementary material for: Deep targeted sequencing of 12 breast cancer susceptibility regions in 4611 women across four different ethnicities
Source: Breast Cancer Res. 2016 Nov 5;18:109. doi: 10.1186/s13058-016-0772-7 (PMC5097387; doi:10.1186/s13058-016-0772-7)

**Figure S1:** Diagram describing the initial QC pipeline for the 12 breast cancer GWAS regions as well as the *TERC* region that was later excluded from analysis since it has not been reported to be associated with breast cancer.


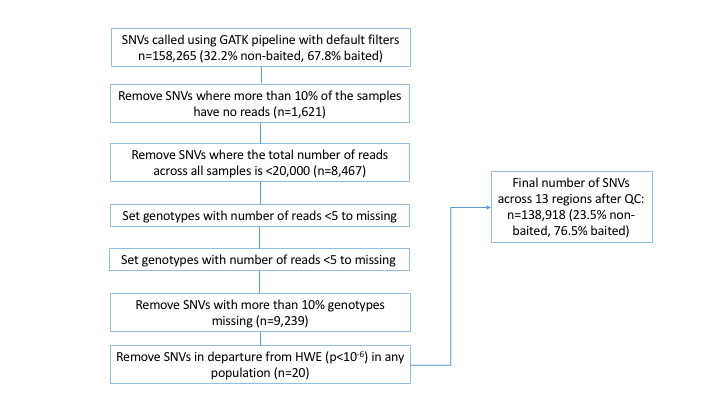

Supplement: Additional file 3: Figure S1. — Diagram describing the initial QC pipeline for the 12 breast cancer GWAS regions as well as the TERC region that was later excluded from analysis because it has not been reported to be associated with breast cancer. (DOCX 135 kb) [file 13058_2016_772_MOESM3_ESM.docx]
