# Supplementary material for: Deep targeted sequencing of 12 breast cancer susceptibility regions in 4611 women across four different ethnicities
Source: Breast Cancer Res. 2016 Nov 5;18:109. doi: 10.1186/s13058-016-0772-7 (PMC5097387; doi:10.1186/s13058-016-0772-7)
Supplement: Additional file 4: Table S3. — Counts and percentages of predictions and PolyPhen-2 scores for nonsynonymous SNVs across all 12 regions. (DOCX 46 kb) [file 13058_2016_772_MOESM4_ESM.docx]

**Table S3.** Counts and percent of predictions and Polyphen-2 Scores for non-synonymous SNVs across all 12 regions

| **Polyphen-2 Prediction** | **Number of SNVs (Number of SNVs also predicted as “None”)** | **Percentage of Predictions** | **Polyphen-2 Score range** |
| --- | --- | --- | --- |
| **Probably damaging** | 427 (1) | 22% | 534 – 616 |
| **Possibly damaging** | 365 (1) | 18% | 284 - 533 |
| **Benign** | 1,060 (3) | 54% | 1 – 283 |
| **Unknown** | 76 (1) | 4% | 1 |
| **None** | 53 | 3% | 617 |
