# Supplementary material for: Deep targeted sequencing of 12 breast cancer susceptibility regions in 4611 women across four different ethnicities
Source: Breast Cancer Res. 2016 Nov 5;18:109. doi: 10.1186/s13058-016-0772-7 (PMC5097387; doi:10.1186/s13058-016-0772-7)
Supplement: Additional file 5: Table S4. — Observed SNVs classified by their impact on gene function as predicted by SnpEff [43]. (DOCX 66 kb) [file 13058_2016_772_MOESM5_ESM.docx]

**Table S4**. Observed SNVs classified by their impact on gene function as predicted by SNPEFF [REF. 43].

|  | **Impact on gene function as predicted by SNPEFF** | | | |
| --- | --- | --- | --- | --- |
| **Variant type** | **High** | **Moderate** | **Low** | **Modifier** |
| STOP CODON GAINED | 48 |  |  |  |
| SPLICE SITE DONOR | 18 |  |  |  |
| SPLICE SITE ACCEPTOR | 12 |  |  |  |
| START CODON LOST | 2 |  |  |  |
| STOP CODON LOST | 1 |  |  |  |
| NON SYNONYMOUS CODING |  | 1983 |  |  |
| SYNONYMOUS CODING |  |  | 1306 |  |
| START CODON GAINED |  |  | 67 |  |
| SYNONYMOUS START CODING |  |  | 1 |  |
| INTRON |  |  |  | 62427 |
| INTERGENIC |  |  |  | 34532 |
| DOWNSTREAM |  |  |  | 15586 |
| TRANSCRIPT |  |  |  | 15937 |
| UPSTREAM |  |  |  | 5360 |
| 3' UTR |  |  |  | 174 |
| 5' UTR |  |  |  | 76 |
| **Total** | **81** | **1983** | **1374** | **134092** |
