# Supplementary material for: Deep targeted sequencing of 12 breast cancer susceptibility regions in 4611 women across four different ethnicities
Source: Breast Cancer Res. 2016 Nov 5;18:109. doi: 10.1186/s13058-016-0772-7 (PMC5097387; doi:10.1186/s13058-016-0772-7)

**Figure S2:** MAF distributions of the 137,530 observed SNVs in the 12 breast cancer GWAS regions across four ethnicities.


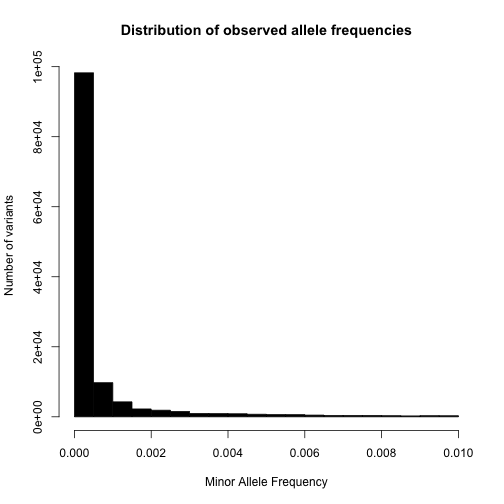

Supplement: Additional file 6: Figure S2. — MAF distributions of the 137,530 observed SNVs in the 12 breast cancer GWAS regions across 4 ethnicities. (DOCX 51 kb) [file 13058_2016_772_MOESM6_ESM.docx]
