# Supplementary figures and images for: Deep targeted sequencing of 12 breast cancer susceptibility regions in 4611 women across four different ethnicities
Source: Breast Cancer Res. 2016 Nov 5;18:109. doi: 10.1186/s13058-016-0772-7 (PMC5097387; doi:10.1186/s13058-016-0772-7)

**Figure S3:** MAF distribution for SNVs observed in both this study and the 1000G project.


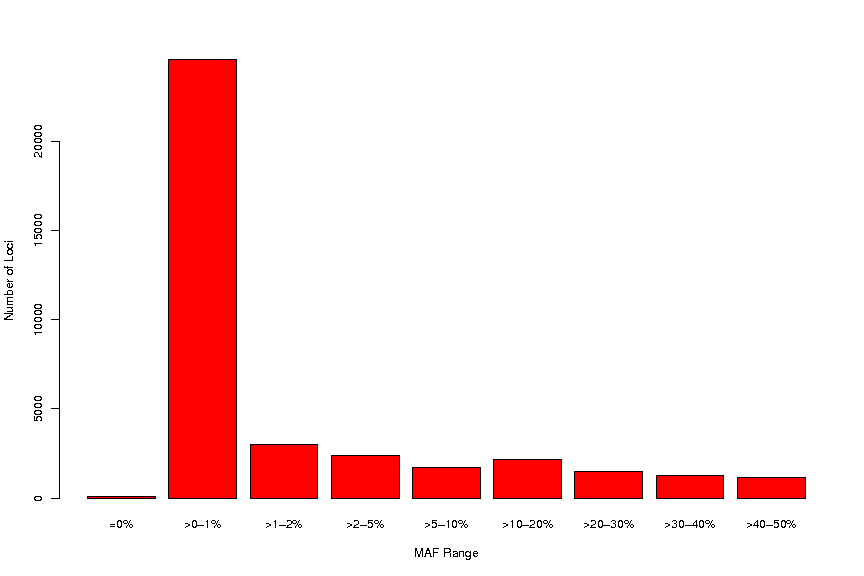

Supplement: Additional file 7: Figure S3. — MAF distribution for SNVs observed in both this study and the 1000 Genomes Project. (DOCX 50 kb) [file 13058_2016_772_MOESM7_ESM.docx]

**Figure S5:** MAF distribution for SNVs in 1000G but not in this study.


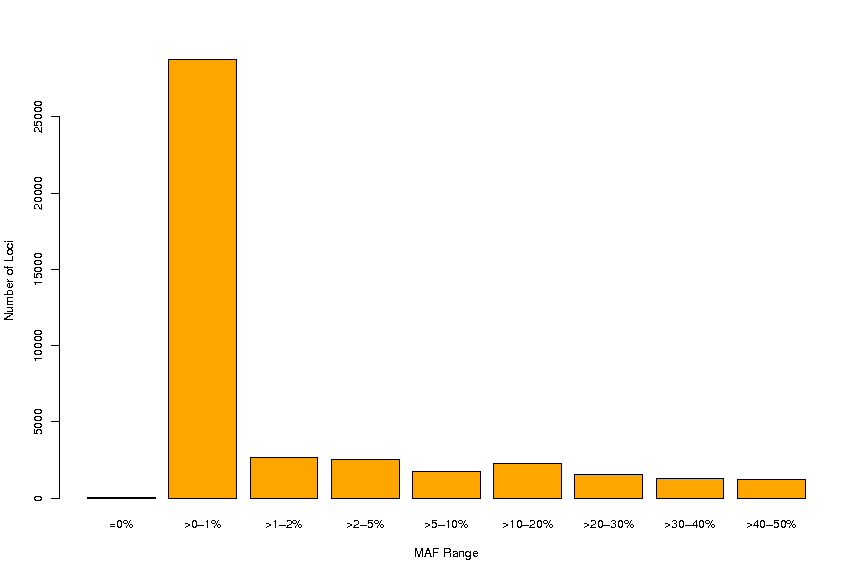

Supplement: Additional file 9: Figure S5. — MAF distribution for SNVs in the 1000 Genomes Project but not in this study. (DOCX 49 kb) [file 13058_2016_772_MOESM9_ESM.docx]
