# Supplementary material for: Deep targeted sequencing of 12 breast cancer susceptibility regions in 4611 women across four different ethnicities
Source: Breast Cancer Res. 2016 Nov 5;18:109. doi: 10.1186/s13058-016-0772-7 (PMC5097387; doi:10.1186/s13058-016-0772-7)

**Figure S4:** MAF distribution for SNVs observed in this study but not in the 1000G project. SNVs observed in this study but not in the 1000G project were almost exclusively very rare (MAF<0.005), and only 0.4% (390 variants) of SNVs not observed in 1000G had a MAF>0.005 compared to 46% (17,160) of variants observed in 1000G


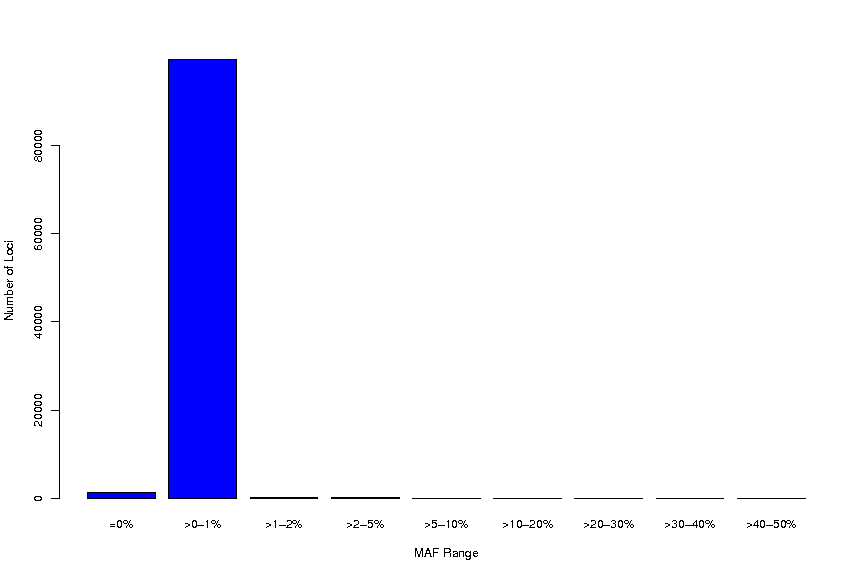

Supplement: Additional file 8: Figure S4. — MAF distribution for SNVs observed in this study but not in the 1000 Genomes Project. SNVs observed in this study but not in the 1000 Genomes Project were almost exclusively very rare (MAF <0.005), and only 0.4 % (390 variants) of SNVs not observed in the 1000 Genomes Project had a MAF >0.005 compared with 46 % (17,160) of variants observed in the 1000 Genomes Project. (DOCX 54 kb) [file 13058_2016_772_MOESM8_ESM.docx]
