# Supplementary material for: Deep targeted sequencing of 12 breast cancer susceptibility regions in 4611 women across four different ethnicities
Source: Breast Cancer Res. 2016 Nov 5;18:109. doi: 10.1186/s13058-016-0772-7 (PMC5097387; doi:10.1186/s13058-016-0772-7)

**Figure S6:** Regional association plots (black, left panel) and posterior probability plots (grey, right panel) by region. Results are based on meta-analysis across all ethnicities. The original index GWAS SNP is highlighted in red.


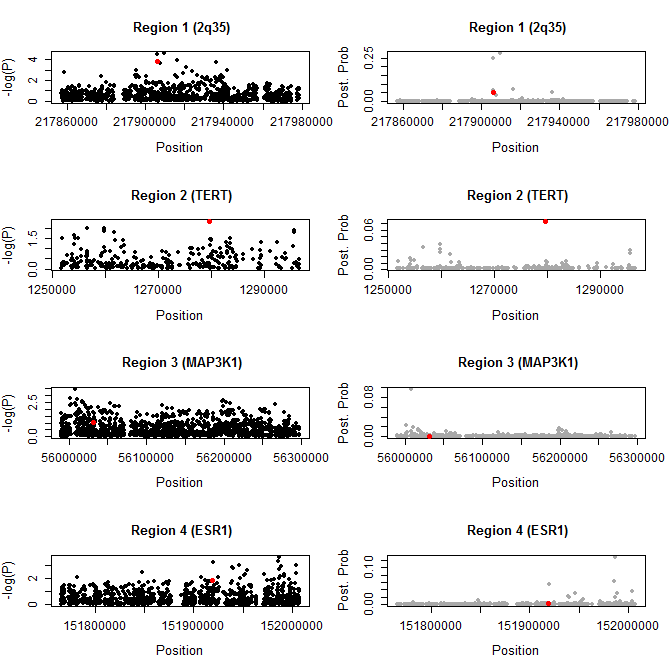


**Figure S6 (continued):**


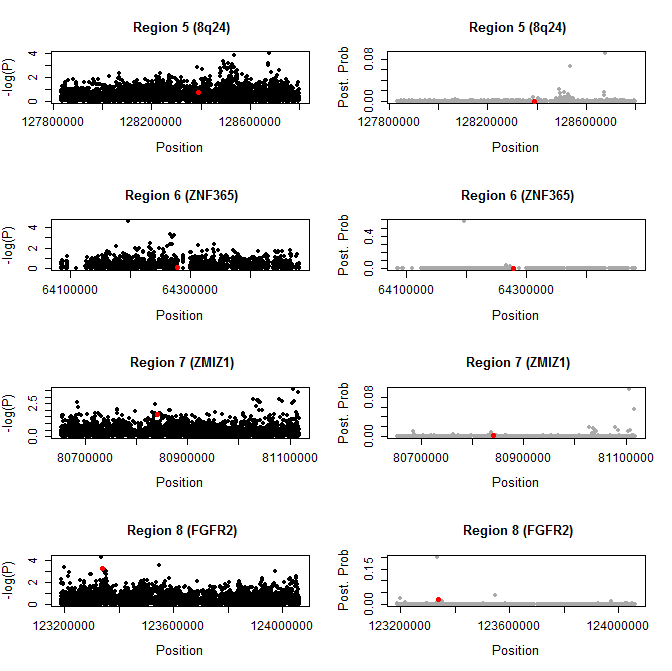


**Figure S6 (continued):**


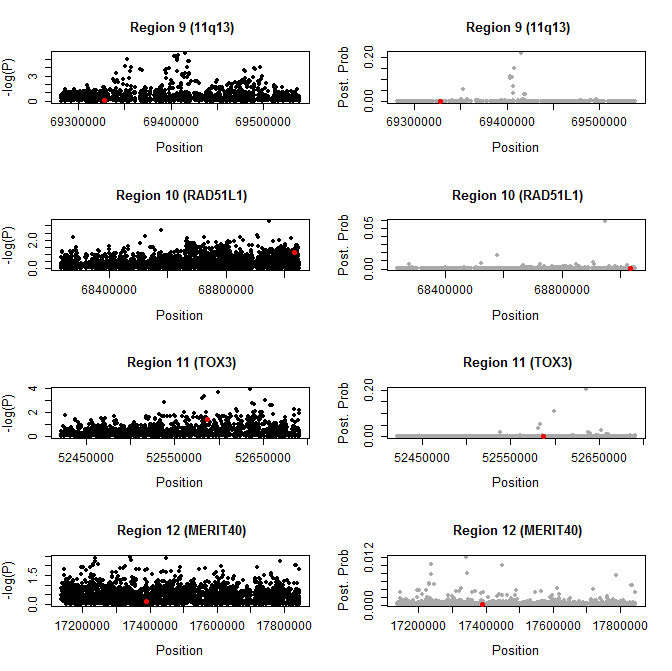

Supplement: Additional file 11: Figure S6. — Regional association plots (black, left panel) and posterior probability plots (gray, right panel) by region. Results are based on meta-analysis across all ethnicities. The original index GWAS SNP is highlighted in red. (DOCX 200 kb) [file 13058_2016_772_MOESM11_ESM.docx]
